# The Positive and Negative Syndrome Scale for Schizophrenia Autism Severity Scale (PAUSS) in a sample of early-onset psychosis

**DOI:** 10.1192/j.eurpsy.2023.954

**Published:** 2023-07-19

**Authors:** J. Suárez Campayo, L. Pina-Camacho, J. Merchán-Naranjo, C. Ordas, V. Cavone, R. Panadero, G. Sugranyes, I. Baeza, J. Castro-Fornieles, E. de la Serna, C. Arango, C. M. Diaz Caneja

**Affiliations:** 1 Department of Child and Adolescent Psychiatry, Institute of Psychiatry and Mental Health, Hospital General Universitario Gregorio Marañón; 2 Instituto de Investigación Sanitaria Gregorio Marañón (IiSGM); 3 Centro de Investigación Biomédica en Red de Salud Mental (CIBERSAM), Instituto de Salud Carlos III; 4 Universidad Complutense de Madrid, Madrid; 5 Institut d’Investigacions Biomèdiques August Pi i Sunyer; 6 Department of Child and Adolescent Psychiatry and Psychology, Hospital Clínic de Barcelona; 7 Universidad de Barcelona, Barcelona, Spain

## Abstract

**Introduction:**

The Positive and Negative Syndrome Scale for Schizophrenia Autism Severity Scale (PAUSS) scale can be derived from the Positive and Negative Schizophrenia Syndrome Scale, enabling an assessment of psychotic and autistic dimensions with a single tool.

**Objectives:**

The aim of the study was to investigate the prevalence of autistic traits and the diagnostic, developmental, clinical, and functional correlates of this phenotype in a sample of early-onset psychosis (onset before age 18 years; EOP).

**Methods:**

Prospective observational 2 year- follow-up study in a sample of young people with a first-episode of EOP. Demographic, perinatal, developmental, cognitive, clinical, and functional data were collected. PAUSS total scores and socio-communication and repetitive behaviors subscores were calculated. We used the proposed cut-off points for adult populations to define prevalence of autistic traits (PAUSS≥30). Subgroups of patients with and without autistic traits were identified based on the total PAUSS terciles. We used the Cronbach’s alpha test to assess the PAUSS internal consistency. Linear mixed models were performed to compare changes in PAUSS during follow-up between diagnostic subgroups [i.e., non-affective psychosis (including schizophrenia and schizophreniform disorder), affective psychosis (including bipolar disorder, schizoaffective disorder and major depressive disorder with psychotic features), and other psychosis (including brief psychotic disorder and psychosis not otherwise specified)]. Developmental, clinical, and functional variables were compared between subgroups with and without autistic traits with logistic regression analysis.

**Results:**

248 patients with PIT were included (age 15.69 ± 1.86 years, 38.65% female). The prevalence of autistic traits in EOP was 7.04%, with significantly higher prevalence in the group of patients with non-affective psychosis (15.20%) than in other diagnostic groups. PAUSS scores significantly decreased over time, with no significant differences in the trajectories of the total PAUSS and its subscores among the three diagnostic subgroups during the 2-year follow-up. The PAUSS showed good internal consistency at all visits (Cronbach’s alpha > 0,88). Patients with autistic traits presented longer duration of untreated psychosis, longer duration of the first inpatient admission, poorer social adjustment in childhood, poorer functionality, greater clinical severity, and poorer response to treatment during follow-up than patients without autistic traits.

**Conclusions:**

The PAUSS is an easy-to-apply tool that can be useful to differentiate psychosis subgroups with worse prognosis.

**Disclosure of Interest:**

J. Suárez Campayo: None Declared, L. Pina-Camacho: None Declared, J. Merchán-Naranjo: None Declared, C. Ordas: None Declared, V. Cavone: None Declared, R. Panadero: None Declared, G. Sugranyes: None Declared, I. Baeza: None Declared, J. Castro-Fornieles: None Declared, E. de la Serna: None Declared, C. Arango Consultant of: Acadia, Angelini, Gedeon Richter, Janssen Cilag, Lundbeck, Minerva, Otsuka, Roche, Sage, Servier, Shire, Schering Plough, Sumitomo Dainippon Pharma, Sunovion and Takeda, C. Diaz Caneja Grant / Research support from: Exeltis and Angelini

